# Neurocognitive outcome in tyrosinemia type 1 patients compared to healthy controls

**DOI:** 10.1186/s13023-016-0472-5

**Published:** 2016-06-29

**Authors:** Willem G. van Ginkel, Rianne Jahja, Stephan C. J. Huijbregts, Anne Daly, Anita MacDonald, Corinne De Laet, David Cassiman, François Eyskens, Irene M. L. W. Körver-Keularts, Philippe J. Goyens, Patrick J. McKiernan, Francjan J. van Spronsen

**Affiliations:** University of Groningen, Beatrix Children’s Hospital, University Medical Center Groningen, 9700 RB Groningen, The Netherlands; University of Leiden, Leiden, The Netherlands; Birmingham Children’s Hospital, Birmingham, UK; University Children’s Hospital Queen Fabiola, Free University of Brussels, Brussels, Belgium; University Hospital Gasthuisberg, University of Leuven, Leuven, Belgium; Queen Paola Children’s Hospital, University of Antwerp, Antwerp, Belgium; Maastricht University Medical Center, Maastricht, The Netherlands

**Keywords:** Tyrosinemia type 1, Neurocognitive outcome, Executive functions, EF, Social cognition, IQ, Working memory, NTBC

## Abstract

**Background:**

Hereditary Tyrosinemia type 1 (HT1) is a rare metabolic disorder caused by a defect in the enzyme Fumarylacetoacetate Hydrolase. Due to this defect, toxic products accumulate which, in turn, cause liver and kidney dysfunction. Treatment with 2-(2-nitro-4-trifluoromethylbenoyl)-1,3-cyclohexanedione (NTBC) and diet has diminished these problems, but recent data indicate that HT1 patients have neurocognitive problems. However, the neuropsychological profile of these patients is unknown. Therefore, this study aimed to investigate this neuropsychological profile by comparing HT1 patients with healthy controls.

**Methods:**

Neurocognitive testing was performed in a heterogeneous group of 19 NTBC and dietary treated HT1 patients (five female, fourteen male; mean age 12.9 ± 4.8 years; range 7.9–23.6 years) and 19 age and gender matched controls (five female, fourteen male; mean age 13.2 ± 4.6 years; range 8.1–24.8 years). IQ scores were estimated and all participants performed the Amsterdam Neuropsychological Tasks, measuring executive functions (inhibition, cognitive flexibility and working memory) and social cognition (face recognition and identification of facial emotions).

**Results:**

HT1 patients showed poorer estimated IQ, executive functioning (working memory and cognitive flexibility), and social cognition compared to healthy controls. Lower IQ scores in HT1 patients were mostly unrelated to scores on executive function- and social cognition tasks and therefore did not account for group differences in these domains. Further analyses within the HT1 patient group (comparing different groups of patients based on the age at diagnosis and the clinical symptoms at diagnosis) did not reveal any significant results. The duration of NTBC treatment was negatively correlated with IQ.

**Conclusions:**

Despite the heterogeneity of the patient group, these data clearly show that IQ, executive functioning and social cognition are affected in HT1 patients, and that IQ screening is not sufficient for cognitive monitoring of these patients. Further research should focus on the underlying pathophysiological mechanisms of these impairments to consequently try to improve treatment strategies.

## Background

Hereditary Tyrosinemia Type 1 (HT1; McKusick 27670) is an inborn error of tyrosine metabolism, caused by a deficiency of the enzyme Fumarylacetoacetate Hydrolase. Due to this defect, toxic products such as maleylacetoacetate, fumarylacetoacetate and succinylacetone are accumulating. These products cause severe liver dysfunction, renal tubulopathy, porphyria like syndrome, cardiomyopathy and hepatocellular carcinoma early in life [1]. In the past, life expectancy was rather low and many patients needed a liver transplantation [2]. This changed after 1992, when a new treatment option became available, called 2-(2-nitro-4-trifluoromethylbenoyl)-1,3-cyclohexanedione (NTBC) [3]. NTBC is an herbicide that blocks the catabolism of tyrosine upstream of the initial enzymatic defect, preventing the formation of the toxic products mentioned before. With this treatment, liver failure, renal tubulopathy, cardiomyopathy and porphyria like syndrome resolved and the life expectancy strongly increased, while the risk of liver cancer largely decreased [4, 5]. Despite the success of NTBC in combination with dietary treatment, current research suggests suboptimal neurocognitive outcome in HT1 patients, including reports of a lower IQ, school problems, and impaired motor control [6–10].

This more or less resembles the history of another metabolic disorder, namely Phenylketonuria (PKU). In PKU, the defect is in the conversion of phenylalanine to tyrosine, leading to an opposite biochemical profile with high blood phenylalanine and somewhat low blood tyrosine concentrations. At first sight, there is no clinical resemblance between PKU and HT1. Untreated PKU is characterized by severely diminished mental development, seizures and behavioural difficulties. However, both diseases have been associated with a suboptimal neurocognitive outcome despite appropriate treatment, which in both cases, involves manipulation of phenylalanine and tyrosine intake and consequently its concentrations [1, 11].

In PKU, different studies have shown that impaired executive functioning (EF) (over and above lower IQ) might aggravate problems with other aspects of cognition (e.g. motor function, visuo-perceptual abilities) [12, 13], and might underlie problems in other domains as well (e.g. social functioning, behaviour problems and quality of life) [14–16]. Next to the impairments in EF, recent research from our group has shown that PKU patients exhibit specific difficulties in social cognition, which, in turn, is also related to social functioning and behaviour problems [17]. This information about EF and social cognition is lacking in HT1. Therefore, this study aimed to investigate the neuropsychological profile, including IQ, core EF and aspects of social cognition, of HT1 patients, and to compare these cognitive outcomes with those of healthy controls.

## Methods

### Patients

Nineteen NTBC and dietary treated HT1 patients (five female, fourteen male; mean age 12.9 ± 4.8 years; range 7.9–23.6 years), with varying clinical histories and treatment strategies (but excluding those who underwent liver transplantation) were studied. They were treated in the Netherlands, UK and Belgium. Phenylalanine and tyrosine restricted diet was started in all patients directly after diagnosis. NTBC treatment was started in 18 patients directly after diagnosis. One patient (Table 1, patient 17) was diagnosed before NTBC became available and therefore NTBC treatment started after 2 years of dietary treatment alone. The diet involved natural protein restriction. Supplementation of all non-phenylalanine and tyrosine amino acids was provided with one of the available amino acid mixtures that contain neither phenylalanine nor tyrosine. Nine patients at some point received phenylalanine supplementation due to phenylalanine concentrations below the lower target limit (<30 μmol/L) [18, 19].

**Table 1:** Detailed patient characteristics

*Time of diagnosis* *(months)*	*Patient*	*Age at diagnosis (months)*	*Current age (years)*	*Duration of NTBC treatment (years)*	*Clinical problems at diagnosis*
*≤2*	*1*	*0,3*	*9,2*	*9,1*	*pre-symptomatically*
	*2*	*0,03*	*14,0*	*14,0*	
	*3*	*0,03*	*15,3*	*15,3*	
	*4*	*0,2*	*8,1*	*8,0*	
	*5*	*0,03*	*15,6*	*15,6*	
	*6*	*0,1*	*12,1*	*12,1*	
	*7*	*0,1*	*10,3*	*10,3*	
	*8*	*0,5*	*10,4*	*10,4*	
	*9*	*0,1*	*8,8*	*8,8*	
	*10*	*1,7*	*10,4*	*10,2*	
*2–6*	*11*	*3,2*	*7,9*	*7,6*	*Acute liver failure and renal tubulopathy*
	*12*	*4,1*	*13,4*	*13,1*	*Acute liver failure*
	*13*	*2,9*	*9,7*	*9,5*	*Acute liver failure*
	*14*	*3,8*	*9,9*	*9,6*	*Acute liver failure*
	*15*	*3,0*	*16,0*	*15,8*	*Acute liver failure, renal tubulopathy and rickets*
	*16*	*5,0*	*17,7*	*17,3*	*Renal tubulopathy, nefromegaly and rickets*
*≥6*	*17*	*8,6*	*23,4*	*20,6*	*Liver cirrhosis*
	*18*	*13,0*	*7,9*	*6,7*	*Liver cirrhosis*
	*19*	*38,7*	*23,6*	*20,4*	*Liver cirrhosis, renal tubulopathy and rickets*

Seven of the 19 healthy controls (five female, fourteen male; mean age 13.2 ± 4.6 years; range 8.1–24.8 years) were friends/siblings of the HT1 patients, while the other 12 healthy controls were age and gender matched only. Some HT1 patients did not complete all tasks. These patients were excluded from statistical analyses of these particular tasks only, together with their age and gender matched controls. Informed consent of all patients and/or parents was obtained and this study was approved by the ethics committees of the different clinical centers.

### Tests

Neuropsychological assessment was performed by WGvG and RJ. Assessments took place at the patients’ clinical treatment center. A broad estimation of IQ was made by using two subtests of the Wechsler Intelligence Scale for Children Third Edition and Fourth edition (WISC) for patients from 7 to 16 years old or the Wechsler Adult Intelligence Scale Third Edition (WAIS) for patients older than 16 years [20–22]. One perceptual reasoning subtest (Block Design) and one vocal comprehension subtest (Vocabulary) of the WISC or WAIS were used to estimate performance and verbal IQ, respectively.

All patients were assessed with the Amsterdam Neuropsychological Tasks [23], measuring EF and social cognition. A standardized protocol was used and a full assessment took up to 3 h to complete. The following EF were measured: inhibitory control (Shifting Attentional Set-Visual (SSV)), working memory (Feature Identification (FI) and Memory Search 2D objects (MS2D)) and cognitive flexibility (SSV), together with the following two social cognition measurements: Face Recognition (FR) and Identification of Facial Emotions (IFE) as described by various study groups [16, 24–26]. In all tasks, both accuracy and reaction time (RT) were measured. To study RT adequately, baseline speed of the patients was measured and subtracted from the RT during the different tasks (or task conditions). In this way, differences in normal RT between HT1 patients and healthy controls were accounted for.

In the SSV task, patients responded to the movements of a coloured square across a bar on the computer screen. They had to respond in accordance (part 1, green stimulus) or opposite (part 2, red stimulus) to the direction of the movement of the coloured square. The action requested of the participants depended on the colour of the square after each movement (green or red) in part 3. The contrast between performance in part 1 and 2 represents inhibitory control, while the contrast between performances in part 1 and 3 represents cognitive flexibility.

The MS2D task was used to measure the ability to maintain an increasing number of stimuli in working memory. Participants had to search a display consisting of four stimuli, each characterized by two specific features (colour and shape), for one (part 1) and one of three target memorized stimuli (part 2). When (one of) the target stimuli was present, participants had to respond with their dominant hand (“yes”-response). If not, a “no”-response had to be given by using their non-dominant hand. The contrast between performances in part 1 and 2 (with higher working memory load) represented the working memory-score.

The FI task also measured working memory. Participants had to scan a display consisting of four 3x3 matrices that included red elements. The target stimulus they had to identify was a (memorized) matrix with three red elements, which were in a fixed position. The other matrices had fewer red elements (easy condition) or equal numbers of elements in different positions (difficult condition). When the display contained the target stimulus, a “yes”-response was required.

In the FR task, the ability to recognize neutral faces was examined. Before each trial, a target face was shown on the display first for 2.5 s. Thereafter four different faces were shown and the participant had to judge if the target individual was presented. If so, a “yes”-response was required, if not, a “no-response” was required. Three task parts were performed for this study. In the first task part, a frontal view of targets and distractors was provided, in the second part, targets were presented “en profile” and in the third task part stimuli were presented upside down.

The IFE task measures the ability to identify emotions using facial expressions. The expressed emotions in the four different task parts were respectively happiness, sadness, anger and fear. When the face on the display showed the target emotion, participants had to use their dominant hand (“yes”-response). If not, participants had to use their non-dominant hand (“no”-response).

### Statistical analyses

Group differences between HT1 and controls were analyzed using Mann–Whitney U tests. Non-parametric tests were used because non-normal and dissimilar distributions (between groups) were observed for a number of neuropsychological outcome measures. In order to find out whether the neuropsychological scores of the patients fell within or outside the normal range (between -2SD and +2SD, with negative numbers indicating better than average accuracy (i.e. lower error rates) or faster than average speed of responding), we calculated z-scores by subtracting the mean task score of all healthy controls from the task score of the patient divided by the standard deviation of the healthy controls.

As we were also interested in whether potential executive function and social cognition impairments would be present over and above (potentially) lower IQ scores in HT1, additional correlations and group differences between HT1 patients and controls were studied after deletion of all patients (and controls) with IQ-scores outside the normal range while maintaining the match on age and gender. To further analyse the heterogeneous HT1 patient group, groups based on the age at diagnosis (≤2 months, 2–6 months, ≥6 months) were compared using the Kruskal Wallis tests. The two-tailed Spearman correlation analyses were performed to further study a possible association between the age at diagnosis and the performance on the neuropsychological tasks. Next to these analyses, pre-symptomatically and symptomatically diagnosed patients and patients presenting with and without liver involvement at diagnosis were compared using Mann–Whitney U tests. Finally, partial correlations between the duration of NTBC treatment and the performance on the neuropsychological tasks while correcting for age were performed. In all statistical tests, a *p*-value of <0.05 was considered statistically significant. Analyses were conducted with the statistical program IBM Statistics 22 (Chicago, Illinois).

## Results

### Participant characteristics

Descriptive analyses of the HT1 patients showed a large variation in age at diagnosis and clinical problems. All patients had received NTBC treatment and a phenylalanine and tyrosine restricted diet for at least 7 years. Ten patients were diagnosed before 2 months of age, pre-symptomatically, by selective screening because of an affected sibling or as a coincidence in the neonatal PKU screening. The mean age of this group was 11.4 years (range 8.1–15.6 years). Six patients were diagnosed between 2 and 6 months of age (mean age 12.5 years, range 8.3–17.7), mostly suffering from acute liver failure and/or renal problems. Three patients were diagnosed after 6 months of age (mean age 18.3 years, range 7.9–23.6 years old), all suffering from cirrhosis, rickets and/or tubular dysfunction (Table 1).

### Neurocognitive outcome

**Fig. 1:**
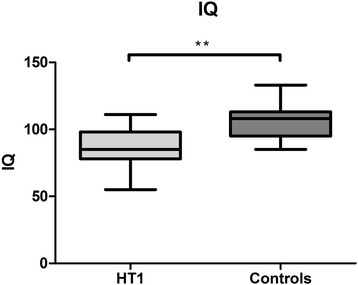
Estimated IQ scores, comparing HT1 patients and controls. *p < 0.05, **p < 0.01

Figure 1 shows estimated IQ scores. The median of the estimated IQ scores in HT1 patients was 85 (range: 55–111), with two patients having an estimated IQ between 55 and 65, one patient with an estimated IQ between 65 and 75, and three patients with an estimated IQ between 75 and 85. All other patients had IQ scores within the normal range of 85–115. Estimated IQ scores of the healthy controls were mostly within the normal range, with four estimated IQ scores above 115. HT1 patients had significantly lower estimated IQ than healthy controls (U =60, *p* < 0.001).

**Fig. 2:**
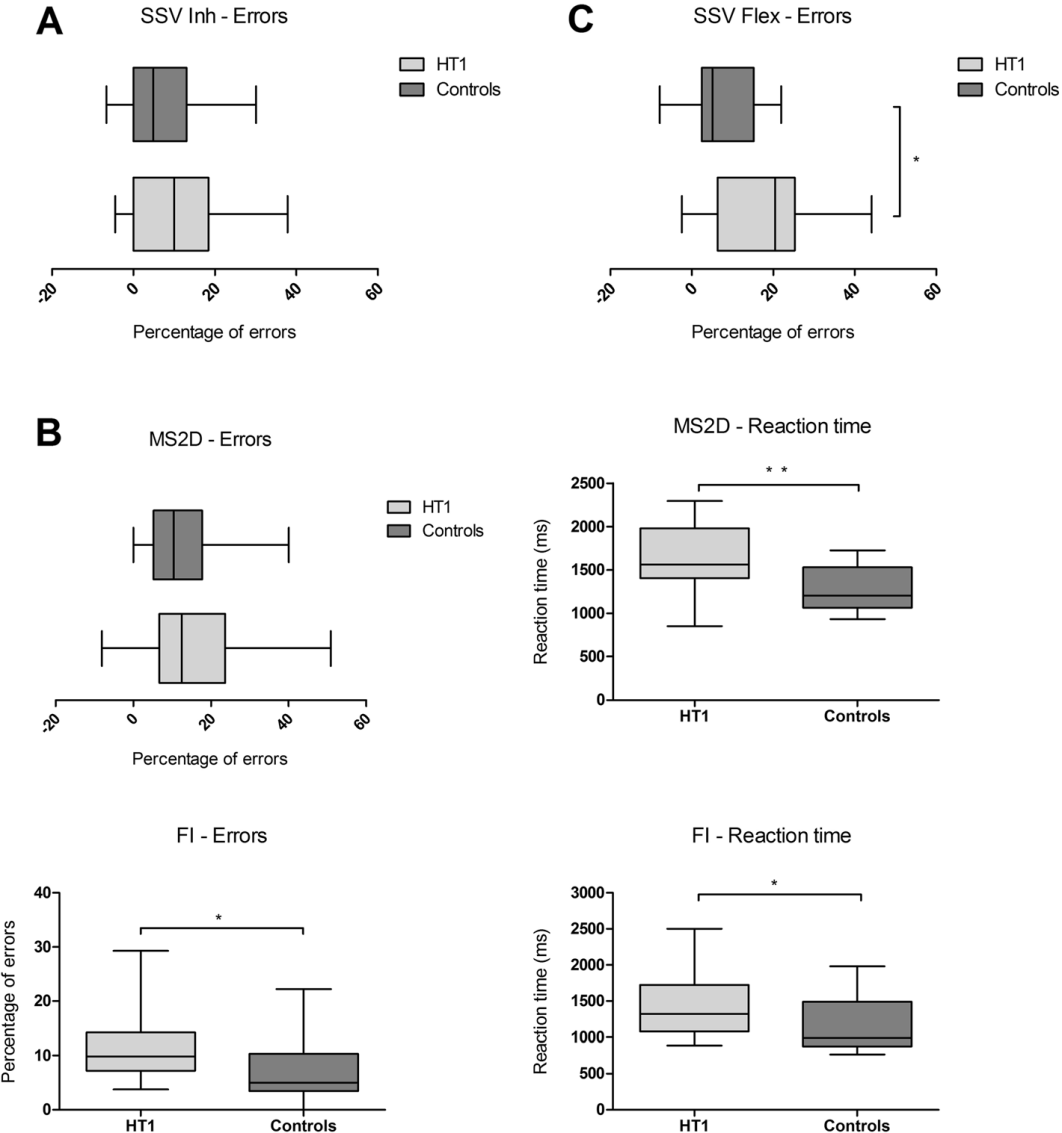
Performance on executive functions comparing HT1 patients and controls. Performance is expressed as percentage of errors and/or reaction time on tasks measuring inhibition **a**, working memory **b** and cognitive flexibility **c** *p < 0.05, **p < 0.01

Figure 2 shows EF scores. Part A shows inhibitory control scores. In the SSV task, there were no significant differences between HT1 patients and healthy controls regarding inhibitory control (Percentage errors: *U* = 153.5, *p* = 0.435; RT: *U* = 153, *p* = 0.435). Part B shows working memory scores for the different groups. In the MS2D task, HT1 patients had a significant longer RT than healthy controls (*U* = 61, *p* = 0.003). In the FI task HT1 patients were also significantly slower than healthy controls (*U* = 84, *p* = 0.013). In addition, HT1 patients made significantly more mistakes than healthy controls in this task (*U* = 98, *p* = 0.044). Part C shows scores on cognitive flexibility. The percentage of errors for HT1 patients was significantly higher compared to healthy controls (*U* = 95, *p* = 0.012). No significant differences for RT were found (*U* = 170, *p* = 0.773).

**Fig. 3:**
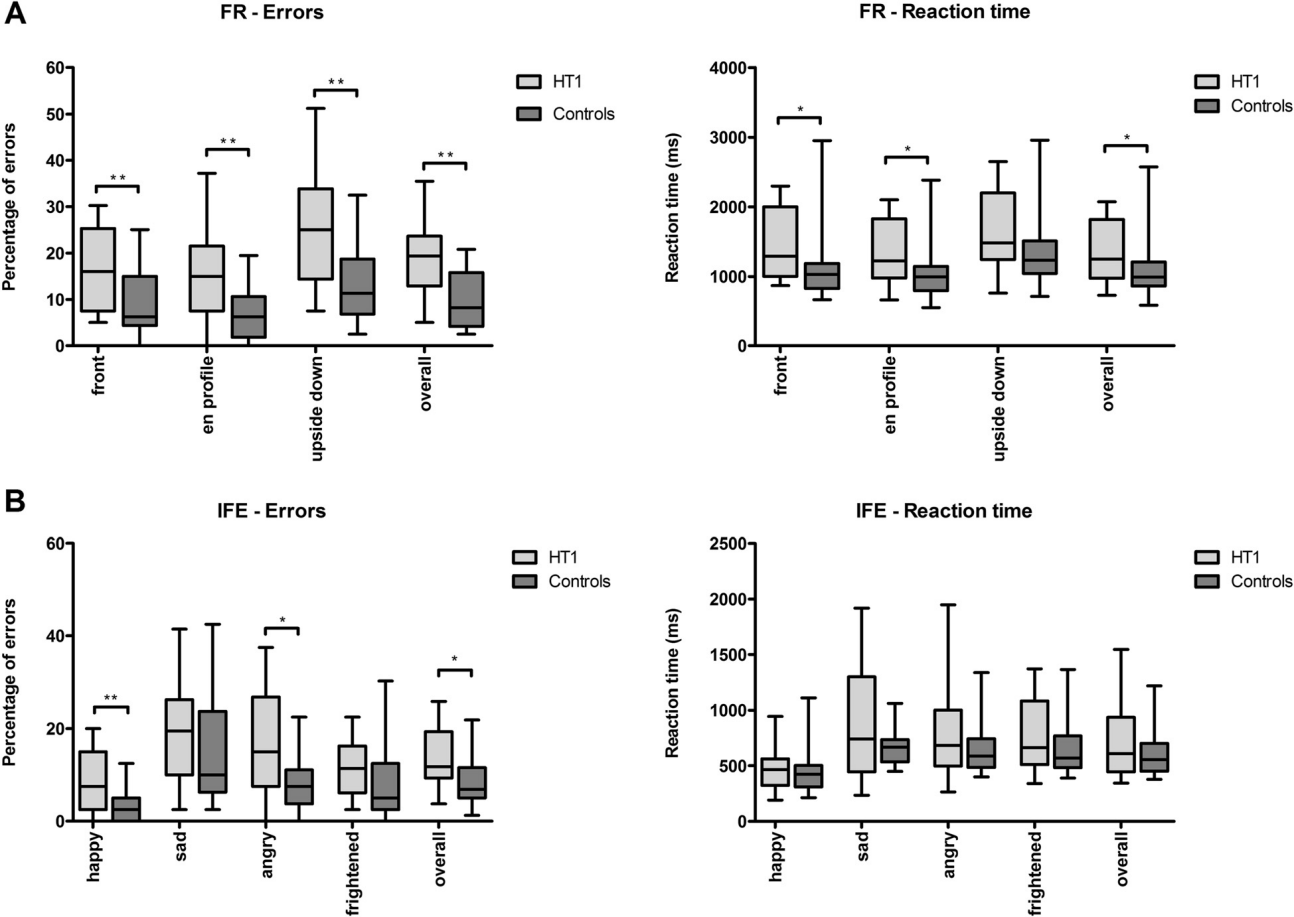
Performance on two different constructs of social cognition expressed as percentage of errors on face recognition **a** and the identification of facial emotions **b** comparing HT1 patients and healthy controls. *p < 0.05, **p < 0.01

Figure 3 shows scores on social cognition. Part A shows scores on FR. The percentage of errors for HT1 patients was overall significantly higher when compared to healthy controls (*U* = 59.5, *p* = 0.001). More precisely, HT1 patients were less accurate than controls on all the different parts of this task. HT1 patients were also significantly slower than healthy controls on this task (*U* = 93, *p* = 0.029). A significant difference in RT was specifically found on the first and second part of the task (*U* = 90, *p* = 0.022 and *U* = 96, *p* = 0.037 respectively). Part B shows scores on the IFE. Overall, the percentage of errors for HT1 patients was significantly higher compared to healthy controls (*U* = 72.5, *p* = 0.012). More specifically, HT1 patients tended to show a higher percentage of errors when they had to identify frightened faces (trend), while a significantly higher percentage of errors was observed for HT1 patients when they had to identify happiness (*U* = 70.5, *p* = 0.009) and anger (*U* = 74.5, *p* = 0.014). In this task, no significant differences in RT were found.

Table 2 shows analyses of Z-scores for the different neuropsychological tasks. Median Z-scores for the HT1-patients varied between tasks and patients scoring outside the normal range were not the same ones in each task. Overall, between 11 % and 32 % of the patients have a Z-score > 2 on one or more of the neuropsychological tasks.

**Table 2:** Z-score analyses on tasks measuring EF and social cognition

	*Age at diagnosis*							
	*<2 months* *N = 8-10*		*2–6 months* *N = 6*		*6 months* *N = 3*		*Overall* *N = 17–19*	
*Tasks*	*RT (s)*	*Errors (%)*	*RT (s)*	*Errors (%)*	*RT (s)*	*Errors (%)*	*RT (s)*	*Errors (%)*
*SSV-inhibition (n = 19)*								
*Median Z-score*	*−0.15*	*−0.11*	*−0.32*	*−0.49*	*0.64*	*0.82*	*−0.15*	*0.27*
*Z-score > +2 (n)*	*1*	*1*	*1*	*1*	*0*	*0*	*2*	*2*
*SSV-Cog. Flex. (n = 19)*								
*Median Z-score*	*0.78*	*1.53*	*−0.17*	*0.67*	*0.54*	*−0.85*	*0.39*	*1.43*
*Z-score > +2 (n)*	*3*	*3*	*0*	*1*	*0*	*1*	*3*	*5*
*MS2D (n = 17)*								
*Median Z-score*	*1.43*	*−0.07*	*1.38*	*−0.35*	*1.10*	*1.54*	*1.12*	*−0.09*
*Z-score > +2 (n)*	*3*	*1*	*2*	*0*	*1*	*1*	*6*	*2*
*FI (n = 18)*								
*Median Z-score*	*0.50*	*0.52*	*0.50*	*0.30*	*0.36*	*1.23*	*0.45*	*0.52*
*Z-score > +2 (n)*	*1*	*1*	*1*	*1*	*1*	*0*	*3*	*2*
*FR-overall (n = 18)*								
*Median Z-score*	*1.14*	*1.80*	*0.34*	*1.36*	*−0.41*	*1.53*	*0.39*	*1.57*
*Z-score > +2 (n)*	*2*	*3*	*0*	*2*	*1*	*1*	*3*	*6*
*IFE-overall (n = 17)*								
*Median Z-score*	*0.29*	*0.67*	*−0.21*	*1.03*	*2.74*	*0.46*	*0.03*	*0.55*
*Z-score > +2 (n)*	*1*	*2*	*0*	*1*	*2*	*0*	*3*	*3*

When only selecting the HT1 patients and age- and gender matched healthy controls with IQ scores within the normal range (85–115), IQ scores were significantly correlated with error percentages FI-working memory (ρ = −0.49, p = 0.027), and SSV-inhibition (ρ = −0.55, *p* = 0.012). Comparing both groups, differences on FI-working memory became non-significant, while differences on SSV-inhibition have not been seen in these analyses, neither in the previous analyses. However, as IQ was not significantly related to any other EF- or social cognition scores, further group differences could not be explained by differences in IQ.

When comparing three different groups of HT1 patients based on the age at diagnosis (<2 months, 2–6 months, >6 months), no significant differences in RT or the percentage of errors in any of the neuropsychological tasks were observed. In addition to this, no significant correlations between the age at diagnosis and task performance were observed. Furthermore, when comparing pre-symptomatically and symptomatically diagnosed patients and when comparing patients with or without liver dysfunction at diagnosis no significant differences in all the tasks measuring IQ, EF and social cognition were found. While controlling for age, the duration of NTBC treatment was significantly negatively correlated with IQ (*r* = −0.51, *p* = 0.046).

## Discussion

This is the first controlled HT1 patient study describing impaired neurocognitive outcome through detailed neuropsychological testing while comparing them with age and gender matched healthy controls. The most important finding was that HT1 patients not only have a suboptimal outcome on IQ, but more specifically also appear to have a suboptimal outcome on EF (working memory and cognitive flexibility) and social cognition (face recognition and the identification of facial emotions). In most of the tasks measuring EF and social cognition, IQ was not correlated with the outcome variables, in which cases IQ could therefore not completely explain the sub optimal outcome on these neuropsychological constructs.

Before discussing the results of the study in more detail, one important methodological limitation of the study has to be addressed. The patient population was very heterogeneous: patients differed in timing of diagnosis and accompanying symptoms. Next to this, dietary and NTBC treatments of HT1 patients are likely to have varied, to some extent, between different clinical centres, and the exact treatment histories could not be taken into account in the present study. Further subgroup analyses, comparing different groups based on the age at diagnosis and clinical symptoms at diagnosis, did not reveal any significant differences, although this may, in part, be explained by the small group sizes.

Our results support and extend previous findings of suboptimal neurocognitive outcome in HT1 [6–10]. Despite using only two subtests of the WISC/WAIS to broadly estimate IQ, clear differences were observed, with HT1 patients performing significantly worse than controls. While IQ is a broad construct measuring intellectual functioning in general, HT1 patients performed worse than controls on most measures of EF and social cognition as well. “Pure” social cognition was measured in the IFE-task and the relatively easy task part 1 of the FR-task. The more difficult parts of this task require, in addition to social cognition, the EF working memory [27]. Our results showed differences in tasks or task parts measuring relatively “pure” social cognition, tasks or task parts measuring “pure” EF and those measuring combinations of social cognition and EF.

In order to investigate the clinical importance of these significant results, Z-scores were calculated to see how many patients score within or outside the normal range. In most of the tasks measuring IQ, EF and social cognition many patients scored below the normal range. This may be interpreted as support for the case that these results have clinical relevance as well.

Several hypotheses have been proposed to explain the suboptimal neuropsychological outcome in HT1 patients, but the mechanism remains unknown. First of all, the fact that neuropsychological impairment was observed after the introduction of NTBC may suggest a possible direct association between these deficiencies and NTBC. However, developing severe liver disease of any cause, especially in infancy, is associated with both a low life expectancy and a risk of long term developmental sequelae and behavioural consequences [28]. Therefore, the low life expectancy in these HT1 patients, if not treated with NTBC, may have masked the impaired neurocognitive outcome associated with liver dysfunction in HT1. Our results did not show significant differences between the patients presenting with and without liver dysfunction, making the latter hypothesis of liver dysfunction in early life causing the developmental problems less likely.

On the other hand the duration of treatment with NTBC was negatively correlated to the IQ. This could indicate a possible direct effect of NTBC on the cognitive outcome. However, NTBC may have induced neurocognitive impairments through its effect on blood phenylalanine and tyrosine concentrations as well. With NTBC treatment, tyrosine concentrations increase even without liver failure, leading to the hypothesis that high tyrosine concentrations may be associated with impaired neurocognitive functioning. High tyrosine concentrations could have a direct toxic effect on the brain, similar to high phenylalanine concentrations in PKU [29, 30]. In Tyrosinemia type II, biochemically characterized by high blood tyrosine and normal blood phenylalanine concentrations, neurocognitive deficiencies have been shown as well. Especially when dietary treatment is started late, these patients are prone to cognitive deficiencies possibly caused by high tyrosine concentrations [31].

In PKU, research has suggested the presence of additional pathophysiological mechanisms, next to phenylalanine toxicity, affecting brain functioning. For example, high blood phenylalanine concentrations may inhibit the transport of the other large neutral amino acids (LNAA) into the brain, due to competitive transport across the blood–brain barrier [30]. Impaired transport of those LNAA into the brain could influence protein and neurotransmitter synthesis in the brain [29, 30]. In HT1 patients, there could also be an impaired influx of LNAAs, but this time caused by an excess of the LNAA tyrosine, which is the metabolic precursor of dopamine. Evidence for abnormalities in neurotransmitter synthesis in HT1 stems from studies showing low cerebral spinal fluid (CSF) concentrations of serotonin and high to normal CSF dopamine concentrations in HT1 patients [32], both possibly related to the high blood tyrosine concentrations. High blood tyrosine concentrations may cause low CSF serotonin levels by blocking the transport of its metabolic precursor, the LNAA tryptophan, across the blood–brain barrier. At the same time, high blood tyrosine concentrations may cause a high influx of tyrosine into the brain causing higher-than-normal CSF dopamine levels. Very low phenylalanine levels in HT1 patients could be important as well. It has been reported that very low phenylalanine levels could have peripheral implications such as eczema and faltering growth, but could also cause developmental delay in HT1 [19]. Low phenylalanine concentrations are sometimes seen in HT1 patients [7, 18, 19]. Thus, both high tyrosine and low phenylalanine concentrations may be important in the cascade ultimately leading to brain dysfunction in HT1. Therefore, future studies have to relate the neuropsychological outcome to treatment parameters such as tyrosine and phenylalanine concentrations throughout life. In addition to this, a direct effect of NTBC on the neurocognitive outcome needs further investigation.

## Conclusions

HT1 patients on average underperformed on the neurocognitive tasks compared to healthy controls when examining IQ, EF, and social cognition. The pathophysiological mechanisms that result in this brain dysfunction are not fully understood yet. Clearly, possible similarities between HT1 and other single amino acid disorders such as PKU could help to elucidate these mechanisms. Future research into HT1 should also compare the neuropsychological outcome between HT1 patients with different treatment regimes. Moreover, future studies should focus on the pathophysiological mechanisms underlying the impairments. This combined knowledge will contribute to further optimization of the treatment strategies for HT1 patients.

## Abbreviations

NTBC, 2-(2-nitro-4-trifluoromethylbenoyl)-1,3-cyclohexanedione; CRF, cerebral spinal fluid; EF, Executive function(s); FR, Face Recognition; FA4L, Focused Attention 4 Letters; FI, Feature Identification; IFE, Identification of Facial Emotions; LNAA, large neutral amino acids; MS2D, Memory Search 2D objects; PKU, Phenylketonuria; RT, Reaction time; SSV, Shifting Attentional Set-Visual; HT1, Tyrosinemia Type 1; WAIS, Wechsler Adult Intelligence Scale Third Edition; WISC, Wechsler Intelligence Scale for Children Third or Fourth Edition
